# Reducing expression of a nitrate‐responsive bZIP transcription factor increases grain yield and N use in wheat

**DOI:** 10.1111/pbi.13103

**Published:** 2019-03-21

**Authors:** Junbo Yang, Meiyue Wang, Wenjing Li, Xue He, Wan Teng, Wenying Ma, Xueqiang Zhao, Mengyun Hu, Hui Li, Yijing Zhang, Yiping Tong

**Affiliations:** ^1^ The State Key Laboratory for Plant Cell and Chromosome Engineering Institute of Genetics and Developmental Biology Chinese Academy of Sciences Beijing China; ^2^ Shanghai Institutes for Biological Sciences Chinese Academy of Sciences (CAS) Shanghai China; ^3^ The Institute for Cereal and Oil Crops Hebei Academy of Agriculture and Forestry Sciences Shijiazhuang China; ^4^ The Innovative Academy of Seed Design Chinese Academy of Sciences Beijing China

**Keywords:** *Triticum aestivum*, leucine zipper transcription factor, NADH‐dependent glutamate synthase, N use efficiency, grain yield

## Abstract

Nitrogen (N) plays critical role in plant growth; manipulating N assimilation could be a target to increase grain yield and N use. Here, we show that ABRE‐binding factor (ABF)‐like leucine zipper transcription factor *TabZIP60* mediates N use and growth in wheat. The expression of *TabZIP60* is repressed when the N‐deprived wheat plants is exposed to nitrate. Knock down of *TabZIP60* through RNA interference (RNAi) increases NADH‐dependent glutamate synthase (NADH‐GOGAT) activity, lateral root branching, N uptake and spike number, and improves grain yield more than 25% under field conditions, while overexpression of *TabZIP60‐6D* had the opposite effects. Further investigation shows TabZIP60 binds to ABRE‐containing fragment in the promoter of *TaNADH‐GOGAT‐3B* and negatively regulates its expression. Genetic analysis reveals that *TaNADH‐GOGAT‐3B* overexpression overcomes the spike number and yield reduction caused by overexpressing *TabZIP60‐6D*. As such, TabZIP60‐mediated wheat growth and N use is associated with its negative regulation on *TaNADH‐GOGAT* expression. These findings indicate that *TabZIP60* and *TaNADH‐GOGAT* interaction plays important roles in mediating N use and wheat growth, and provides valuable information for engineering N use efficiency and yield in wheat.

## Introduction

The macronutrient nitrogen (N) is essential for plant growth, and is a primary constituent of the nucleotides and proteins. N is the most widely used fertilizer in promoting crop productivity. Plants absorb N from the soil mainly in the form of nitrate and ammonium. Once entered the cells, these inorganic N compounds are assimilated into amino acids, and thus N assimilation represents a physiological process of the utmost importance for plant growth and development (Mokhele *et al*., 2012). A better understanding for the regulation of N uptake and assimilation is vital for breeding crops with improved yield and N use efficiency (Kong *et al*., 2013; Masclaux‐Daubresse *et al*., 2010; Xu *et al*., 2012).

Plants mainly depend on nitrate transporters and ammonium transporters in the root system for N uptake from the soil. The NRT1/NPF family includes low‐affinity nitrate transporters, whereas the NRT2 family encodes high‐affinity nitrate transporters. NRT1/NPF and NRT2 families are involved in sensing nitrate and in regulating primary nitrate responses (Krapp *et al*., 2014; Xu *et al*., 2012). In rice, the nitrate transporters from both NRT1/NPF and NRT2 families have been shown to increase N uptake and yield under sufficient and low N conditions (Chen *et al*., 2016a; Fan *et al*., 2016; Hu *et al*., 2015; Wang *et al*., 2018b). Quantitative trait locus (QTL) mapping has revealed the linakge between yield and N assimilation genes in wheat and maize (Hirel *et al*., 2001; Kichey *et al*., 2006; Quraishi *et al*., 2011). Modulating the expression of N assimilation genes has successfully improved crop yield. The transgenic expression of *AlaAT* (alanine aminotransferase) under the control of the *antiquitin* gene promoter significantly enhances yield in canola and rice (Good *et al*., 2007; Shrawat *et al*., 2008). Glutamine synthetase (GS)/glutamate synthase (GOGAT) cycle is the first step in the assimilation of inorganic N onto carbon (C) skeletons for the production of amino acids. This has prompted geneticists and plant breeders to find ways in improving yield and N use efficiency by manipulating GS/GOGAT. Knocking out *OsGS1.1* inhibits rice growth and grain filling (Tabuchi *et al*., 2005), whereas *ZmGS1.3* (*Gln1‐3*) overexpression in maize increases grain number (Martin *et al*., 2006). *TaGS2* overexpression in wheat increases N uptake, N allocation to grains and yield in wheat (Hu *et al*., 2018). As such, manipulating N uptake and assimilation genes can increase crop productivity.

A number of genes have been found to regulate N uptake and assimilation, and some of them have been used to engineering crops with improved yield and N use efficiency. AtNLP7 (NIN‐LIKE PROTEIN 7) plays a key role in nitrate signalling and regulate the expression of many N transporters and assimilation genes in Arabidopsis (Konishi and Yanagisawa, 2013). Overexpressing *AtNLP7* in Arabidopsis increased plant biomass under both low N and high N conditions, and overexpressing *AtNLP7* in tobacco (*Nicotiana tabaccum*) also improved plant growth and N use (Yu *et al*., 2016). Overexpression of a maize transcription factor (TF) *Dof1* (DNA BINDING WITH ONE FINGER) in Arabidopsis increased the expression of phosphoenolpyruvate carboxylase (PEPC) and several genes involved in the tricarboxylic acid cycle and thereby produce more carbon skeletons for the assimilation of N (Yanagisawa, 2004). And *ZmDof1* also has been shown to increase carbon flow towards N assimilation and to improve N assimilation and growth of rice under low N conditions (Kurai *et al*., 2011). In wheat, the nuclear factor Y TF *TaNFYA1‐6B* and NAC (NAM, ATAF1/2 and CUC2) TF *TaNAC2‐5A* significantly promote root growth and enhance the expression of *NRT1* and *NRT2* families, and thus, increase N uptake and grain yield in wheat (He *et al*., 2015; Qu *et al*., 2015). *TaNAC2‐5A* also has been shown to positively regulate *TaGS2* expression (He *et al*., 2015). The Green Revolution greatly increased crop yield, and the semi‐dwarfism of green revolution varieties is conferred by mutant alleles at the *Rht* in wheat and *SD1* in rice. However, mutant *sd1* and *Rht* alleles inhibit N uptake (Li *et al*., 2018). A recent study in rice shows that higher expression of the *Growth‐Regulating Factor 4* (*GRF4*) TF promotes ammonium uptake and yield of Green Revolution varieties (Li *et al*., 2018). The rice *DEP1* encodes a G protein γ subunit and plays a key role in controlling panicle architecture (Huang *et al*., 2009). The dominant allele at the *DEP1* locus (*dep1‐1*) is a gain‐of‐function mutation, and can increase transcript levels of key genes associated with ammonium uptake and assimilation (*OsAMT1.1*,* OsGS1.2* and *OsNADH‐GOGAT1*), N uptake and grain yield at moderate levels of N fertilization, compared to the NIL with the *DEP1* allele (Sun *et al*., 2014). The rice mutant *abc1‐1*, a weak mutant allele of ferredoxin‐dependent GOGAT (*Fd‐GOGAT*), displays a typical N‐deficient syndrome (Yang *et al*., 2016). Moreover, the loss of function of *ARE1*, a suppressor of *abc1‐1*, could partially rescue the phenotype of *abc1‐1* and enhance yield (Wang *et al*., 2018a). Thus, identification of genes regulating N uptake and assimilation genes could help achieve higher yield and efficient N use.

The basic leucine zipper (bZIP) TFs are involved in plant development, environmental signalling and stress response (Droge‐Laser *et al*., 2018). The Arabidopsis bZIP TFs comprises 78 members, which have been divided into 13 groups (Droge‐Laser *et al*., 2018), and the members from Groups A, D, H and S have been reported to regulate N use. The Group S member *AtbZIP1* is a master regulator in propagating N nutrient signals (Para *et al*., 2014). The Group D members *AtTGA1* and *TGA4* function as important regulators of nitrate response (Alvarez *et al*., 2014). The Group H members *AtHY5* and *AtHYH* regulate nitrate and ammonium transporters, and nitrate and nitrite reductase (Jonassen *et al*., 2008, 2009; Chen *et al*., 2016b; Gangappa and Botto, 2016). The Group A member *AtABI5* is involved in regulating C/N cross talk and nitrate‐induced inhibition on lateral development (Signora *et al*., 2001; Lu *et al*., 2015). Up‐to‐date, the roles of bZIP TFs in regulating N use have not been explored in wheat. Although a number of bZIP TFs have been used to engineer crops with improved tolerance to abiotic and biotic stresses, it has not been reported in the application of bZIP TFs in engineering wheat with improved yield and N use. The wheat bZIP TF *TabZIP60* (GenBank Accession No. KJ806560.1) was first reported in enhancing multiple abiotic stresses. Overexpression of *TabZIP60* in *Arabidopsis* confers drought and cold resistance, and increases plant sensitivity to ABA (Zhang *et al*., 2015). TabZIP60 shows close relation with the members in Group A of bZIP family in *Arabidopsis*, and many members of this group play critical roles in ABA signalling (Droge‐Laser *et al*., 2018). Here, we found that *TabZIP60* is a negative regulator in wheat growth and N use. Overexpression of *TabZIP60* inhibits wheat growth, whereas reducing *TabZIP60* expression through RNAi interference improves grain yield and N use efficiency partially by up‐regulating *TaNADH‐GOGAT* expression.

## Results

### Nitrate represses *TabZIP60* expression

**Figure 1:**
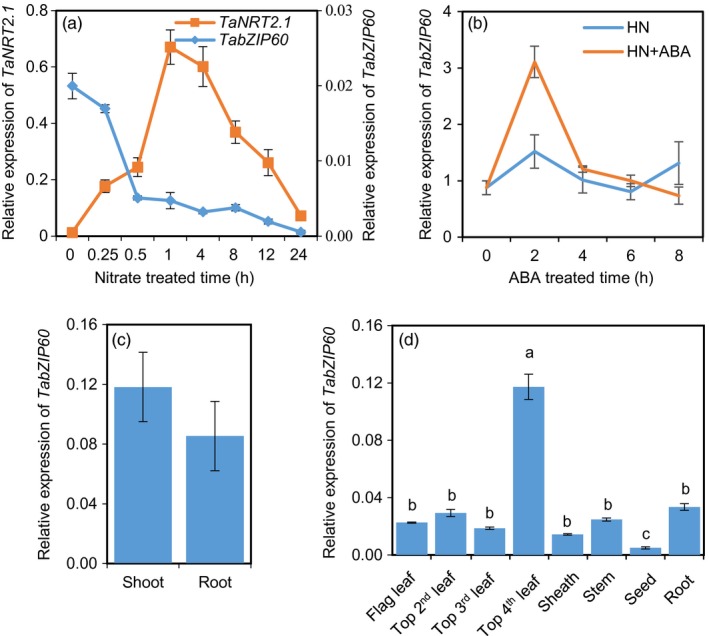
Expression patterns of *TabZIP60*. (a) Responses of *TabZIP60* and *TaNRT2.1‐6B* expression to nitrate in the roots. Wheat seedlings deprived of N for 2 days were exposed to a nutrient solution containing 2 mm NO_3_^−^ for the indicated times. (b) Response of *TabZIP60* to exogenous ABA in roots. HN, 2 mm NO_3_^−^; HN + ABA, 2 mm NO_3_^−^ + 50 μm ABA. (c) Expression levels of *TabZIP60* in shoots and roots at the seedling stage. (d) Expression of *TabZIP60* in different organs of wheat plants at 14 days after flowering under field conditions. The relative expression levels were normalized to the expression of *TaACTIN*. Data are expressed as the mean ± SE of three replicates.

To dissect the regulation mechanism of N assimilation, we performed RNA‐seq analysis of wheat seedling roots in response to nitrate. We found that the expression of *TabZIP60* was significantly reduced 15 min after the N‐deprived wheat seedlings were exposed to 2 mm nitrate. This result was confirmed by using quantitative RT‐PCR. When the N‐deprived wheat seedlings were exposed to 2 mm nitrate for up to 24 h, *TabZIP60* transcript abundance in roots rapidly decreased during the first half hour and then gradually decreased (Figure 1a). However, the expression of *TaNRT2.1*, which encodes a known nitrate‐inducible nitrate transporter (Cai *et al*., 2007), significantly increased within the first hour and then decreased (Figure 1a). This result suggested that nitrate inhibited *TaBZIP60* expression. Phylogenetic analysis reveals that TabZIP60 is most closely related to the ABRE‐binding factor AtABF2 followed by AtABF3 and 4 (Figure S1), which play crucial roles in ABA signalling (Kang *et al*., 2002; Uno *et al*., 2000). Then we examined the expression patterns of *TabZIP60* in response to ABA, and observed that it was induced by exogenous ABA in roots (Figure 1b), further confirming the previous reported ABA‐induction of this gene (Zhang *et al*., 2015). Tissue‐specific analysis revealed that the expression of *TabZIP60* was similar in the shoots and roots of seedlings (Figure 1c). Furthermore, at 14 days post‐anthesis (DPA), *TabZIP60* transcripts were detected in all the investigated organs, with the highest levels observed in the older leaves (top fourth leaves), and the lowest in developing seeds (Figure 1d). These results suggested that *TabZIP60* might participate in wheat N signalling and ABA signalling simultaneously, and *TabZIP60* may function in both shoots and roots during vegetative and reproductive development.

### Reducing *TabZIP60* expression increases grain yield and N uptake

**Figure 2:**
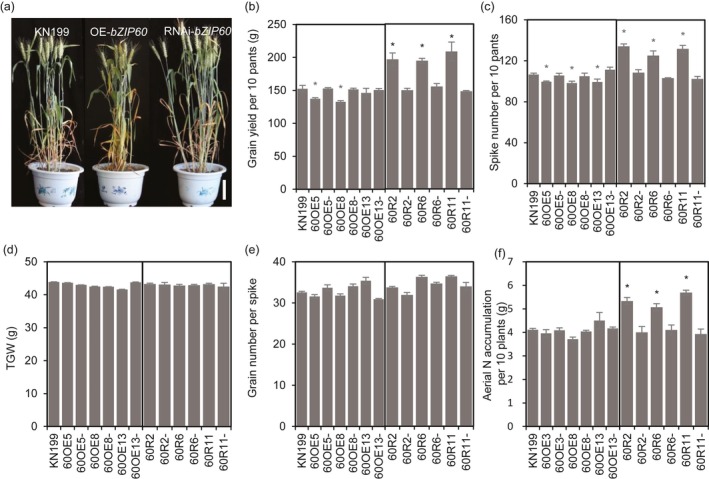
Agronomic traits of *TabZIP60* transgenic lines in the 2016–2017 growing season. (a) Images of transgenic lines and KN199. Bar = 10 cm. (b) Grain yield per 10 plants. (c) Spike number per plant. (d) 1000‐grain weight (TGW). (e) Grain number per spike. (f) Aerial N accumulation per 10 plants (ANA). 60OE5, 60OE8 and 60OE13 indicate overexpression lines; 60OE5‐, 60OE8‐ and 60OE13‐ are the corresponding azygous control lines. 60R2, 60R6 and 60R11 indicate the knockdown lines; 60R2‐, 60R6‐ and 60R11‐ indicate the corresponding azygous control lines. The data are expressed as the mean ± SE of four replicates. *Indicates that the difference between the transgenic line and its corresponding azygous line is significant at *P *<* *0.05.

To explore the roles of *TabZIP60* in mediating wheat growth and N use, we generated *TabZIP60‐6D* overexpression lines and *TabZIP60* RNAi lines. Compared with the wild‐type KN199 and azygous control lines (NC, negative controls), *TabZIP60* expression in the shoots and roots was significantly higher in the *TabZIP60‐6D* overexpression lines (Figure S2A), but significantly lower in the *TabZIP60* RNAi lines (Figure S2B). These results indicated that *TabZIP60‐6D* was successfully overexpressed in the *TabZIP60‐6D* overexpression lines and knocked down in the *TabZIP60*‐RNAi lines. In field experiments, the RNAi lines showed a significant increase (25.1%–39.6%) in grain yield than the wild‐type and the corresponding azygous controls, which was due to an increase (21.3%–28.6%) in spike number in 2016–2017 growing seasons (Figure 2b,c). In contrast, the overexpression lines of *TabZIP60‐6D* had lower grain yield compared to the wild‐type and the corresponding azygous controls in both the 2015–2016 and 2016–2017 growing seasons, primarily due to a decrease in spike number (Figures 2b,c and S3A,B). Both overexpression and knock down of *TabZIP60* did not significantly alter the 1000‐grain weight (TGW) and grain number per spike (Figures 2d,e and S3C,D). We also measured aerial N accumulation (ANA) at maturity, and found that knock down of *TabZIP60* shows a significant higher ANA compared to the wild‐type, while *TabZIP60‐6D* overexpression lines shows a similar ANA level with the wild‐type (Figure 2f).

### Reducing *TabZIP60* expression promotes root growth and N use at seedling stage

**Figure 3:**
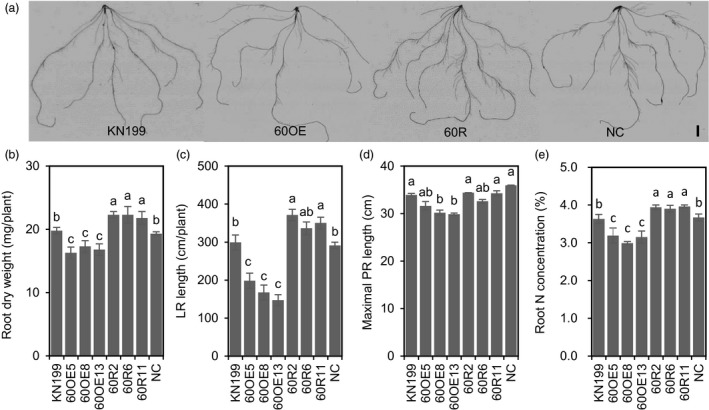
*TabZIP60* affects root growth of wheat seedlings. The 7‐day‐old germinated seedlings of wild‐type (KN199), *TabZIP60‐6D* overexpression lines (60OE5, 8, 13), *TabZIP60* RNAi lines (60R2, 6, 11) and azygous control lines (NC) separated from T1 plants were grown for 14 days in nutrient solutions that contained 2 mm NO_3_^−^. (a) Root images of *TabZIP60* transgenic lines. Bar = 20 mm. (b) Root dry weight. (c) Lateral root (LR) length. (d) Maximal primary root (PR) length. (e) Root N concentration. The data are expressed as the mean ± SE, *n *≥* *3. The data of NC are presented as the mean of all azygous control lines. Different letters in (b) to (d) indicate statistically significant differences at *P *<* *0.05.

To understand the mechanism of reducing *TabZIP60* expression in enhancing N uptake, we then checked the roles of *TabZIP60* in mediating root growth. In a hydroponic culture, we observed stronger root system in the *TabZIP60* knockdown lines (Figure 3a), as the knockdown lines had higher root dry weight (RDW, Figure 3b), longer lateral root (LR) length (Figure 3c) than the wild‐type and NC, but maximal primary root (PR) length did not significantly altered (Figure 3d). These results indicated reducing *TabZIP60* expression enhances root system by promoting lateral root growth, which may facilitate N uptake. We then detected the N concentrations in the roots of *TabZIP60* transgenic lines. The N concentration in roots of the *TabZIP60* knockdown lines was significantly higher than that of KN199 and NC (Figure 3e), indicating reducing *TabZIP60* expression may improve N use. In contrast to the *TabZIP60* knockdown lines, the overexpression lines had the opposite effects on RDW, LR length and root N concentration (Figure 3a–c,e). These results suggest that *TabZIP60* plays a negative role in mediating root growth and N use.

### *TabZIP60* has impact on the loss of leaf N during grain filling

To understand the role of *TabZIP60* in mediating N use, we measured N concentrations in 10 aerial parts at anthesis, 14 DPA (days post‐anthesis) and maturity in the 2016–2017 growing season (Figure S4A–C). The 10 aerial parts included spike, stem and leaf blade, and sheath of four leaves from the flag leaf to top fourth leaf. All the *TabZIP60‐6D* overexpression lines, the *TabZIP60* RNAi lines and the wild‐type KN199 exhibited a decrease in N concentrations in stem, leaf blades and sheathes with grain filling (Figure S4B–J), indicating a N loss in these organs during grain filling. The most apparent difference between the wild‐type and transgenic lines was observed for the N concentrations in leaves at 14 DPA. At anthesis, the significant differences between the wild‐type and transgenic lines were only detected in leaf blade and sheath of the top fourth leaf (Figure S4I,J). At 14 DPA, the significant differences between the wild‐type and transgenic lines were observed for the N concentrations in leaf blades and sheathes of all the four investigated leaves, and these differences disappeared at maturity (Figure S4C–J). These results indicated that *TabZIP60* regulates leaf N level and time‐course of leaf N loss during grain filling. Measurement of grain N concentration did not find the significant difference between the wild‐type and transgenic lines (Table S1).

### Reducing *TabZIP60* expression increases NADH‐GOGAT activity

**Figure 4:**
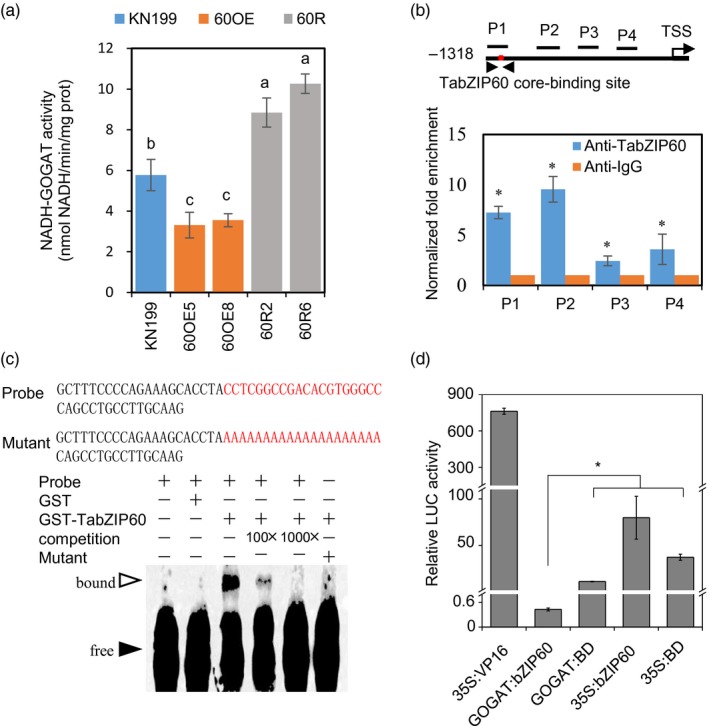
TabZIP60 regulates the expression of *TaNADH‐GOGAT‐3B*. (a) TaNADH‐GOGAT activity in roots of the wild‐type and *TabZIP60* transgenic lines. KN199, wild‐type; 60OE5 and 8, *TabZIP60‐6D* overexpression line; 60R2 and 60R6, *TabZIP60* RNAi line. Different letters above the column indicate statistically significant differences at *P *<* *0.05. (b) ChIP‐qPCR assay of TabZIP60 binding to *TaNADH‐GOGAT‐3B* promoter *in vivo*. TSS, transcription start site. (c) EMSA of TabZIP60 binding to the P1 fragment from (b) *in vitro*. (d) TabZIP60‐6D represses the promoter activity of *TaNADH‐GOGAT‐3B* in a transient expression assay using *Arabidopsis* leaves. The data are expressed as the mean ± SE (*n *≥* *3). *Indicates statistically significant differences at *P *<* *0.05.

Previous studies have suggested that ABFs bind to ABA‐responsive elements ABREs (Izawa *et al*., 1993; Foster *et al*., 1994). Since TabZIP60 is most closely related to ABFs (Figure S1), we analysed the promotor sequences of N assimilation genes, and found that the *TaNADH‐GOGAT* promoter contains several ABREs (Figure S5A). Enzyme activity assay demonstrated that the NADH‐GOGAT activity in roots of the overexpression lines was reduced to 57.4%–61.5% of wild‐type level, whereas that of the RNAi lines was 53.2%–77.8% higher (Figure 4a). As such, *TaNADH‐GOGAT* may be the target gene of TabZIP60. We then checked whether TabZIP60 could bind to the *TaNADH‐GOGAT* promoter through chromatin immunoprecipitation (ChIP)‐qPCR analysis. ChIP‐qPCR revealed the binding enrichment of TabZIP60 to the promoter of *TaNADH‐GOGAT‐3B* (*GOGATpro*, Figure 4b). Furthermore, we conducted electrophoretic mobility shift assay (EMSA) to investigate whether TabZIP60 could bind to the 57‐bp P1 fragment in Figure 4b, which was strongly enriched in ChIP‐qPCR and contained putative ABRE elements. The results showed that TabZIP60 bound to the biotin‐labelled P1 fragment (Figure 4c). In addition, the binding disappeared using the unlabelled P1 fragment as competition, and the mutated P1 probe with a mutation in the putative ABRE element was not bound by TabZIP60 (Figure 4c). These results indicated that TabZIP60 binds to *GOGATpro* in an ABRE‐dependent manner. We next performed a transient expression assay to test whether TabZIP60 had any transcriptional regulatory effect on *GOGATpro*. In a luciferase (LUC) reporter assay system, firefly LUC was used as a reporter. The *LUC* gene was driven by *GOGATpro*, which was connected by five copies of the GAL4 binding element. The GAL4 DNA‐binding domain (BD) could bind to the GAL4 element. We fused BD with *TabZIP60*. The results showed that *GOGATpro::TabZIP60* system had lower LUC activity than control samples (*GOGATpro::GAL‐BD*,* 35S::bZIP60* and *35S::GAL‐BD*; Figure 4d). These results suggest that TabZIP60 directly binds to *GOGATpro* and represses the transcription of *TaNADH‐GOGAT‐3B*.

We also analysed whether *TabZIP60* affected the expression of primary N assimilation genes. Nitrate and ammonia are the major N resources for plant uptake. Nitrate is first reduced to ammonia before its incorporation into organic forms. In primary N assimilation, ammonia is assimilated into glutamine (Gln) and Glu through the GS/GOGAT cycle; Gln and Glu can then be used to form Asp and asparagine (Asn) through the activity of aspartate aminotransferase (AAT) and asparagine synthetase (AS) (Coruzzi, 2003). In addition, GS, GOGAT or glutamate dehydrogenase (GDH) has been implicated in N re‐assimilation (Coruzzi, 2003). After analysing gene expression in the roots of hydroponically grown wheat seedlings, we found that overexpressing *TabZIP60‐6D* inhibited the expression of the genes encoding NR, cytosolic GS (GS1), Fd‐GOGAT, NADH‐GOGAT, GDH and AS (Figure S6A–H).

### Overexpressing *TaNADH‐GOGAT* increases grain yield

**Figure 5:**
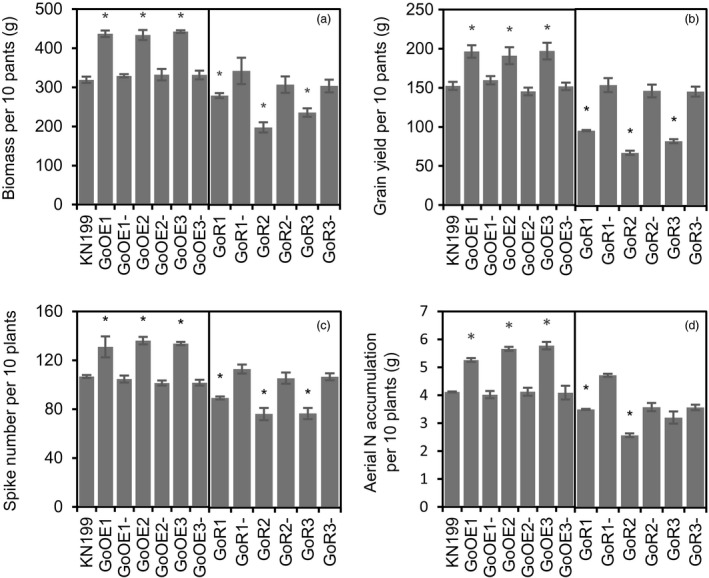
Yield and N use‐related traits of the *TaNADH‐GOGAT* transgenic lines in the field experiment in the 2016–2017 growing season. (a) Biomass per 10 plants. (b) Grain yield per 10 plants. (c) Spike number per 10 plants. (d) Aerial N accumulation per 10 plants (ANA). GoOE1, GoOE2 and GoOE3 indicated positive overexpression lines, GoOE1‐, GoOE2‐ and GoOE3‐ indicated the azygous lines of GoOE1, GoOE2 and GoOE3 respectively. GoR1, GoR2 and GoR3 indicated positive RNAi lines, GoR1‐, GoR2‐ and GoR3‐ indicated the azygous lines of GoR1, GoR2 and GoR3 respectively. Data are means ± SE of three replicates. *Indicates the difference between the positive transgenic line and its corresponding azygous line was significant at *P *<* *0.05.

**Figure 6:**
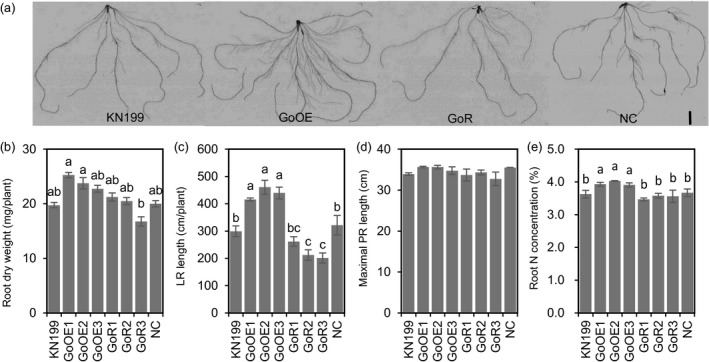
*TaNADH‐GOGAT* affects root growth and N use in wheat seedlings. The 7‐day‐old germinated seedlings of wild‐type (KN199), *TaNADH‐GOGAT‐3B* overexpression lines (GoOE1, GoOE2 and GoOE3), *TaNADH‐GOGAT* RNAi lines (GoR1, GoR2 and GoR3) and azygous control lines (NC) were grown for 14 days in nutrient solutions that contained 2 mm NO_3_^−^. (a) Root images of *TaNADH‐GOGAT* transgenic lines. Bar = 20 mm. (b) Root dry weight. (c) Lateral root (LR) length. (d) Maximal primary root (PR) length. (e) Root N concentration. Data are means ± SE, *n *≥* *3. Data of NC are presented as mean value of all the azygous control lines. Different letters in (b) to (d) indicate statistically significant differences at *P *<* *0.05.

**Figure 7:**
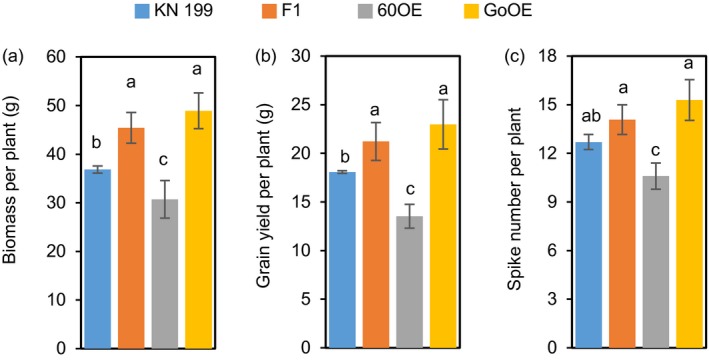
*TaNADH‐GOGAT‐3B* overexpression overcomes the yield reduction by overexpressing *TabZIP60‐6D*. (a) Biomass per plant. (b) Grain yield per plant. (c) Spike number per plant. KN199, wild‐type; 60OE,* TabZIP60‐6D* overexpression lines; GoOE,* TaNADH‐GOGAT‐3B* overexpression lines; 60OE × GoOE (F_1_), F_1_ between 60OE and GoOE cross. Seven F_1_ lines were developed using three 60OE lines (60OE5, 8 and 13) and three GoOE lines (GoOE1, 2 and 3). The data are expressed as the mean ± SE (*n *=* *7). Different letters above the columns indicate statistically significant differences at *P *<* *0.05.

Since *TaNADH‐GOGAT* acts downstream of *TabZIP60*, we further asked how *TaNADH‐GOGAT* mediated wheat growth and N use. We successfully generated *TaNADH‐GOGAT* transgenic lines through overexpression and RNAi approaches (Figure S2C, D). The field experiments showed a better performance of the agronomic traits of overexpression lines in both the 2015–2016 and 2016–2017 growing seasons (Figures 5a and S7). The grain yields of the overexpression lines were increased 16.6%–26.8% compared to the wild‐type in 2015–2016 growing seasons (Figure S7A), and 18.7%–23.9% compared to their corresponding azygous controls in the next year (Figure 5b). The statistical results showed the increment primarily caused by an increase in spike number (11.2%–13.2% in 2015–2016 and 20%–25.5% in 2016–2017; Figures 5c and S7D). In contrast, the RNAi lines showed a significant reduction in grain yield than the wild‐type and the corresponding azygous controls in the two growing seasons, which was mainly due to a reduction in spike number (Figures 5c and S7D). We then measured ANA of the transgenic lines at maturity. The results shows that overexpression lines show a significant higher grain ANA compared to the corresponding azygous controls, while RNAi lines show a lower ANA compared to the corresponding azygous controls (Figure 5d). These results indicated that the expression level of *TaNADH‐GOGAT* is positively related to grain yield and N uptake. A hydroponic culture of seedlings also revealed the positive roles of *TaNADH‐GOGAT* in root growth and root N concentrations (Figure 6a–e). Therefore, overexpression *TaNADH‐GOGAT* could improve wheat growth and N use. After investigated the yield of the F_1_ lines from a cross between *TabZIP60‐6D* and *TaNADH‐GOGAT‐3B* overexpression lines, we found that the F_1_ plants showed higher values for biomass, grain yield and spike number than the wild‐type and *TabZIP60* overexpression lines, but were similar to that of the *TaNADH‐GOGAT‐3B* overexpression lines (Figure 7). These results suggest that *TabZIP60* mediates wheat growth at least partially by regulating *TaNADH‐GOGAT*.

## Discussion

Our current study presents evidence on the roles of *TabZIP60* in mediating wheat growth and N use. Firstly, reducing *TabZIP60* expression greatly increased lateral root branching and root N concentration of wheat seedlings, and improved grain yield and N uptake (ANA) under field conditions, while overexpressing *TabZIP60* had the opposite effects. Secondly, altering *TabZIP60* expression changed leaf N level and time‐course of leaf N loss during grain filling. In the study on the mechanism underlying the regulation of *TabZIP60* on N use, we observed the inhibitory effects of *TabZIP60* on the expression of a number of genes in primary N assimilation and re‐assimilation. ChIP‐qPCR, EMSA and luciferase reporter assays clearly show that TabZIP60 binds to the promoter of *TaNADH‐GOGAT‐3B*, possibly in an ABRE element‐dependent manner, and negatively regulates *TaNADH‐GOGAT‐3B* expression. This negative regulation is in line with the fact that knock down of *TabZIP60* increases NADH‐GOGAT activity in the roots, whereas overexpressing *TabZIP60* reduces. Applying nitrate to the N‐deprived wheat seedlings inhibits *TabZIP60* expression, but induces *TaNADH‐GOGAT* expression in the roots. This contrasting response of *TabZIP60* and *TaNADH‐GOGAT* to nitrate can be explained, at least partially, by the negative regulatory effect of TabZIP60 on *TaNADH‐GOGAT*.

GOGAT plays essential roles in primary N assimilation and re‐assimilation (Coruzzi, 2003) and presents in a small gene family in plants. Our analysis of the reference sequence from the wheat variety Chinese spring (http://plants.ensembl.org/Triticum_aestivum/Info/Index) shows that each of the three sub‐genomes in wheat has one *Fd‐GOGAT* on group 2 chromosomes and one *NADH‐GOGAT* on group 3 chromosomes. Investigating the phenotypes of *TaNADH‐GOGAT* overexpression and RNAi lines has revealed the positive roles of these genes in root N concentration and root growth at the seedling stage, as well as in aerial N accumulation, spike number, grain number per spike, and biomass and grain yield at maturity. Similar results have been reported in rice by characterizing *OsNADH‐GOGAT1* mutants. A previous study has shown that *TaNADH‐GOGAT* is orthologous to *OsNADH‐GOGAT1* on chromosome 3 in rice (Quraishi *et al*., 2011). *OsNADH‐GOGAT1* is mainly expressed in the roots and is important for primary ammonium assimilation in roots, root growth, the development of active tiller number and spikelet weight (Lu *et al*., 2011; Tamura *et al*., 2010; Yamaya *et al*., 2002).

Our current study demonstrated that reducing the expression of *TabZIP60* increased not only grain yield but also N uptake. These increasing effects were associated with the increased NADH‐GOGAT activity by reducing *TabZIP60* expression, as several phenotypes of the *TabZIP60* RNAi and overexpression lines resemble those of *TaNADH‐GOGAT* overexpression and RNAi lines respectively. Both *TabZIP60* knockdown and *TaNADH‐GOGAT‐3B* overexpression resulted in increasing lateral root branching, root N concentration, spike number, biomass and grain yields, whereas the opposite effects were observed in both *TabZIP60‐6D* overexpression lines and *TaNADH‐GOGAT* knockdown lines. Genetic analysis showed that overexpressing *TaNADH‐GOGAT‐3B* overcomes the reduced spike number, and biomass and grain yield in the overexpression lines of *TabZIP60‐6D*; this result further support the claim that negative control of *TabZIP60* on wheat yield is associated with its negative control on NADH‐GOGAT expression. *TabZIP60* has been reported a positive role in tolerance to multiple abiotic stresses (Zhang *et al*., 2015). In Arabidopsis, overexpression of *AtABF2* retards growth, but enhances tolerance to multiple abiotic stresses, whereas knockout of *AtABF2* enhances growth of seedlings (Kim *et al*., 2004). These results suggest that *TabZIP60*‐related ABFs may mediate the balance between stress tolerance and growth. Considering the fluctuating environments in wheat growing season, further research is needed to investigate the effects of reducing *TabZIP60* on stress tolerance.

TabZIP60 is most closely related to ABFs in *Arabidopsis* (Figure S1). The expression of *TabZIP60* is induced by exogenous ABA treatment in wheat (Figure 1B), as has been shown previously in wheat (Zhang *et al*., 2015). Overexpression of *TabZIP60* in *Arabidopsis* increases tolerance to multiple abiotic stress and the sensitivity to ABA (Geng *et al*., 2018; Zhang *et al*., 2015). These results indicate a role of *TabZIP60* in ABA signalling and stress tolerance. The ABA signalling pathways have been well documented, from the ABA receptors, the protein kinases, to the nuclear TFs (Raghavendra *et al*., 2010). In the nucleus, the TF ABI5 and related ABFs are the key targets of the protein kinases involved in ABA signalling, and the ABFs bind to the ABRE element, in concert with other transcriptional regulators, provide the ABA‐responsive transcription (Droge‐Laser *et al*., 2018; Raghavendra *et al*., 2010). Although ABI5 is known to control lateral root development in response to nitrate, and C/N cross talk, it is not reported to directly control the expression of N uptake and assimilation genes. As such, TabZIP60‐related ABF(s) may connect ABA signalling with primary N assimilation. Our study also suggested the potential of manipulating ABA signalling components in increasing crop productivity. This idea is supported by the recent study that knockout of ABA receptors via CRISPR/Cas9 technology effectively promotes rice growth and increases grain yield by 31% under field conditions (Miao *et al*., 2018).

## Experimental procedures

### Materials

Winter wheat (*Triticum aestivum*) variety Kenong 199 (KN199) was used to amplify and isolate gene sequences and generate transgenic lines. To generate overexpression lines, the cDNAs of *TabZIP60‐6D* and *TaNADH‐GOGAT‐3B* were inserted into the *pUbi‐163* vector, resulting in *pUbi::TabZIP60‐6D* and *pUbi::TaNADH‐GOGAT‐3B* constructs. To generate knockdown lines, the sequences characterized for *TabZIP60* and TaNADH‐GOGAT were inserted into a *pUbi‐RNAi* vector, resulting in *pUbi::TabZIP60‐RNAi* and *pUbi::TaNADH‐GOGAT‐RNAi* constructs. The above constructs were then transformed into immature embryos of wheat variety KN199 as described elsewhere (Shan *et al*., 2014). The primers used for vector construction are listed in Table S2.

### Hydroponic culture

Seedlings of the wild‐type KN199 and T3 transgenic lines and their azygous control plants separated from T1 plants were used in the hydroponic cultures. Seeds were surface sterilized with 1.5% H_2_O_2_ for 10 h and washed five times with sterile water, and then germinated at 20 ± 1 °C for 7 days. Subsequently, the seedlings were transferred to plastic boxes containing 13 L of nutrient solution. The nutrient solution (normal N conditions) and growth conditions are as described by Shao *et al*. (2017). After growing for 2 weeks, the roots and shoots were harvested separately. The root morphological parameters were measured using Win‐RHIZO software (Regent Instruments Canada, Inc., Ottawa, ON, Canada) as described elsewhere (Ren *et al*., 2012). The total N concentrations in the dried root samples were measured using a semi‐automated Kjeldahl method (Kjeltec Auto 1030 Analyzer; Tecator).

### Field experiments

The wild‐type KN199, transgenic lines and their azygous control lines were used in the field experiments at the experimental station of the Institute for Cereal and Oil Crops, Hebei Academy of Agriculture and Forestry Sciences, Hebei province, China. For the *TabZIP60‐6D* and *TaNADH‐GOGAT‐3B* overexpression lines and *TaNADH‐GOGAT* RNAi lines, T3 and T4 generations were used in the 2015–2016 and 2016–2017 wheat growing seasons respectively. For *TabZIP60* RNAi lines, the T3 generation was used in the 2016–2017 growing season. Fertilizer application (high N conditions) was as described elsewhere (Shao *et al*., 2017). In the 2015–2016 growing season, three replications were used. For each genotype in each replicate, 20 seeds were sown in one 2‐m‐long row, and the rows were spaced 23 cm apart. The yield‐related traits (grain yield, spike per plant, grain number per spike and 1000‐grain weight) of 15 representative plants in each replicate were recorded. In the 2016–2017 growing season, four biological replicates were used. For each genotype in each replicate, 40 seeds were sown in one 2‐m‐long row, and the rows were spaced 23 cm apart. To evaluate N distribution, the leaf, leaf sheath, stem, spike and grain samples were separately collected from 10 randomly selected culms at anthesis, 14 days post‐anthesis (DPA) and maturity. At maturity, the yield‐related traits of 10 representative plants in each replicate were recorded. The total N concentrations plant samples were measured using a semi‐automated Kjeldahl method (Kjeltec Auto 1030 Analyzer; Tecator).

### Quantitative real‐time PCR

Total RNA was extracted from wheat fresh samples using the Plant RNeasy Kit TRIzol reagent (Thermo Fisher Scientific, Waltham, MA). First‐strand cDNA was synthesized from 2 μg of DNase I‐treated total RNA using murine leukaemia virus reverse transcriptase (Promega, Madison, WI). Quantitative real‐time PCR analysis was performed with a LightCycler 480 engine (Roche, Mannheim, Germany) using the LightCycler480 SYBR Green I Master Mix (Roche, Mannheim, Germany). The relative expression levels were normalized to the expression of *TaACTIN* gene. The primers used for quantitative real‐time PCR are detailed in Table S2.

### NADH‐GOGAT activity assay

The roots of 2‐week‐old KN199 plants cultured by hydroponic experiments were used for the analysis of NADH‐GOGAT activity. Analysis of NADH‐GOGAT enzyme activity was performed as described previously (Anderson *et al*., 1989) with minor modifications. A NADH‐GOGAT activity kit (Comin biotechnology, Suzhou, China) was used for the detection of NADH‐GOGAT activity. Briefly, plant extracts were prepared by grinding 0.1 g fresh roots in 800 μL of cold extraction buffer (buffer 1) at 4 °C, and then cleared by centrifugation at 16 000 × *g* for 10 min at 4 °C, followed by collection of the supernatants on ice. Then, we use the BCA assay as described previously (Bainor *et al*., 2011) for determination of protein concentration. To assay NADH‐GOGAT activity, 20 μL plant extracts was mixed with 180 μL reaction mixture (buffer 2), and then transferred into a quartz cuvette incubated in a DU^®^ 800 Nucleic Acid/Protein Analyzer (Beckman Coulter, US) for the Kinetics/Time Run. The NADH‐GOGAT activity [nmol NADH/min/mg prot] was measured spectrophotometrically by recording the rate of NADH oxidation at 340 nm.

### ChIP‐qPCR

Two‐week‐old KN199 plants cultured hydroponically were used for the ChIP‐qPCR. Anti‐TabZIP60 was ordered from the Ab‐Mart Company (Shanghai, China). ChIP assays were performed as described previously (Bowler *et al*., 2004). The primers used for RT‐qPCR are listed in Table S2.

### EMSA

The full‐length CDS of *TabZIP60* was cloned into the *pGEX‐4T‐1* and transferred to *Escherichia coli* BL21 (*Transseta*) to obtain the fusion protein. EMSAs were performed using the LightShift Chemiluminescent EMSA Kit (Thermo Fisher Scientific, Shanghai, China). The probes used for EMSA are listed in Table S2.

### Luciferase reporter assay system

The primers used for luciferase reporter assay system are listed in Table S2. Methods from Chen lab (http://sourcedb.genetics.cas.cn/zw/zjrck/200907/t20090721_2130989.html).

### Statistical analysis

Statistical analysis was conducted using one‐way ANOVA was performed with the SPSS17.0 package for Windows (SPSS, Inc., Chicago, IL).

## Conflict of interest

The authors declare they have no conflict of interest.

## Authors’ contributions

Y.T., J.Y., W.T., X.H., W.M., X.Z. and H.L. designed the experiments and analysed the data. J.Y. performed most experiments, with the assistance of M.W. and M.H. Y.T., J.Y. and Y.T. wrote the paper. All authors have read and commented on the paper.

## Supporting information


**Figure S1** Phylogenetic analysis of TabZIP60 and the bZIP members from *Arabidopsis*.
**Figure S2** Relative expression levels of *TabZIP60* and *TaNADH‐GOGAT* in shoots and roots of their corresponding transgenic lines.
**Figure S3** Yield‐related traits of the *TabZIP60‐6D* overexpression lines in the field experiment in the 2015–2016 growing season.
**Figure S4** N concentrations (%) in aerial organs in *TabZIP60* transgenic lines and KN199 during grain filling.
**Figure S5** Promotor sequence and expression analysis of *TaNADH‐GOGAT*
**Figure S6** Relative expression levels of genes involved in N assimilation.
**Figure S7** Yield‐related traits of the *TaNADH‐GOGAT* transgenic in the field experiment in the 2015–2016 growing season.

**Table S1** Yield and N use‐related traits of the transgenic lines.
**Table S2** Primers used in this study.

## References

[pbi13103-bib-0001] Alvarez, J.M. , Riveras, E. , Vidal, E.A. , Gras, D.E. , Contreras‐López, O. , Tamayo, K.P. , Aceituno, F. *et al*, *et al* (2014) Systems approach identifies *TGA1* and *TGA4* transcription factors as important regulatory components of the nitrate response of *Arabidopsis thaliana* roots. Plant J. 80, 1–13. 25039575 10.1111/tpj.12618

[pbi13103-bib-0002] Anderson, M.P. , Vance, C.P. , Heichel, G.H. and Miller, S.S. (1989) Purification and characterization of NADH‐glutamate synthase from alfalfa root nodules. Plant Physiol. 90, 351–358. 16666762 10.1104/pp.90.1.351 PMC1061721

[pbi13103-bib-0003] Bainor, A. , Chang, L. , McQuade, T.J. , Webb, B. and Gestwicki, J.E. (2011) Bicinchoninic acid (BCA) assay in low volume. Anal. Biochem. 410, 310–312. 21078286 10.1016/j.ab.2010.11.015

[pbi13103-bib-0004] Bowler, C. , Benvenuto, G. , Laflamme, P. , Molino, D. , Probst, A.V. , Tariq, M. and Paszkowski, J. (2004) Chromatin techniques for plant cells. Plant J. 39, 776–789. 15315638 10.1111/j.1365-313X.2004.02169.x

[pbi13103-bib-0005] Cai, C. , Zhao, X.Q. , Zhu, Y.G. , Li, B. , Tong, Y.P. and Li, Z.S. (2007) Regulation of the high‐affinity nitrate transport system in wheat roots by exogenous abscisic acid and glutamine. J. Integr. Plant Biol. 49, 1719–1725.

[pbi13103-bib-0006] Chen, J. , Zhang, Y. , Tan, Y. , Zhang, M. , Zhu, L. , Xu, G. and Fan, X. (2016a) Agronomic N‐use efficiency of rice can be increased by driving *OsNRT2.1* expression with the *OsNAR2.1* promoter. Plant Biotechnol. J. 14, 1705–1715. 26826052 10.1111/pbi.12531 PMC5066696

[pbi13103-bib-0007] Chen, X. , Yao, Q. , Gao, X. , Jiang, C. , Harberd, N.P. and Fu, X. (2016b) Shoot‐to‐root mobile transcription factor HY5 coordinates plant carbon and N acquisition. Curr. Biol. 26, 640–646. 26877080 10.1016/j.cub.2015.12.066

[pbi13103-bib-0008] Coruzzi, G.M. (2003) Primary N‐assimilation into amino acids in *Arabidopsis* . Arabidopsis Book, 2, e0010. 22303223 10.1199/tab.0010 PMC3243381

[pbi13103-bib-0009] Droge‐Laser, W. , Snoek, B.L. , Snel, B. and Weiste, C. (2018) The *Arabidopsis* bZIP transcription factor family‐an update. Curr. Opin. Plant Biol. 45, 36–49. 29860175 10.1016/j.pbi.2018.05.001

[pbi13103-bib-0010] Fan, X. , Tang, Z. , Tan, Y. , Zhang, Y. , Luo, B. , Yang, M. , Lian, X. *et al* (2016) Overexpression of a pH‐sensitive nitrate transporter in rice increases crop yields. Proc. Natl Acad. Sci. USA, 113, 7118–7123. 27274069 10.1073/pnas.1525184113 PMC4932942

[pbi13103-bib-0011] Foster, R. , Izawa, T. and Chua, N.H. (1994) Plant bZIP proteins gather at ACGT elements. FASEB J. 8, 192–200. 8119490 10.1096/fasebj.8.2.8119490

[pbi13103-bib-0012] Gangappa, S.N. and Botto, J.F. (2016) The multifaceted roles of *HY5* in plant growth and development. Mol. Plant, 9, 1353–1365. 27435853 10.1016/j.molp.2016.07.002

[pbi13103-bib-0013] Geng, X. , Zang, X. , Li, H. , Liu, Z. , Zhao, A. , Liu, J. , Peng, H. *et al*, *et al* (2018) Unconventional splicing of wheat *TabZIP60* confers heat tolerance in transgenic *Arabidopsis* . Plant Sci. 274, 252–260. 30080611 10.1016/j.plantsci.2018.05.029

[pbi13103-bib-0014] Good, A.G. , Johnson, S.J. , De Pauw, M. , Carroll, R.T. , Savidov, N. , Vidmar, J. , Lu, Z. *et al* (2007) Engineering N use efficiency with alanine aminotransferase. Can. J. Bot. 85, 252–262.

[pbi13103-bib-0015] He, X. , Qu, B. , Li, W. , Zhao, X. , Teng, W. , Ma, W. , Ren, Y. *et al* (2015) The nitrate inducible NAC transcription factor TaNAC2‐5A controls nitrate response and increases wheat yield. Plant Physiol. 169, 1991–2005. 26371233 10.1104/pp.15.00568 PMC4634051

[pbi13103-bib-0016] Hirel, B. , Bertin, P. , Quillere, I. , Bourdoncle, W. , Attagnant, C. , Dellay, C. , Gouy, A. *et al* (2001) Towards a better understanding of the genetic and physiological basis for nitrogen use efficiency in maize. Plant Physiol. 125, 1258–1270. 11244107 10.1104/pp.125.3.1258 PMC65606

[pbi13103-bib-0017] Hu, B. , Wang, W. , Ou, S. , Tang, J. , Li, H. , Che, R. , Zhang, Z. *et al* (2015) Variation in *NRT1.1b* contributes to nitrate‐use divergence between rice subspecies. Nat. Genet. 47, 834–838. 26053497 10.1038/ng.3337

[pbi13103-bib-0502] Hu, M.Y. , Zhao, X.Q. , Liu, Q. , Hong, X. , Zhang, W. , Zhang, Y.J. , Sun, L.J. *et al* (2018) Transgenic expression of plastidic glutamine synthetase increases nitrogen uptake and yield in wheat. Plant Biotechnol J. 16, 1858–1867. 29577547 10.1111/pbi.12921 PMC6181211

[pbi13103-bib-0018] Huang, X. , Qian, Q. , Liu, Z. , Sun, H. , He, S. , Luo, D. , Xia, G. *et al* (2009) Natural variation at the *DEP1* locus enhances grain yield in rice. Nat. Genet. 4, 494–497. 10.1038/ng.352 19305410

[pbi13103-bib-0019] Izawa, T. , Foster, R. and Chua, N.H. (1993) Plant bZIP protein DNA binding specificity. J. Mol. Biol. 230, 1131–1144. 8487298 10.1006/jmbi.1993.1230

[pbi13103-bib-0020] Jonassen, E.M. , Lea, U.S. and Lillo, C. (2008) HY5 and HYH are positive regulators of nitrate reductase in seedlings and rosette stage plants. Planta, 227, 559–564. 17929051 10.1007/s00425-007-0638-4

[pbi13103-bib-0021] Jonassen, E.M. , Sandsmark, B.A. and Lillo, C. (2009) Unique status of NIA2 in nitrate assimilation: NIA2 expression is promoted by HY5/HYH and inhibited by PIF4. Plant Signal. Behav. 4, 1084–1086. 20009559 10.4161/psb.4.11.9795 PMC2819521

[pbi13103-bib-0022] Kang, J.‐Y. , Choi, H.‐I. , Im, M.‐Y. and Kim, S.Y. (2002) Arabidopsis basic leucine zipper proteins that mediate stress‐responsive abscisic acid signaling. Plant Cell, 14, 343–357. 11884679 10.1105/tpc.010362 PMC152917

[pbi13103-bib-0023] Kichey, T. , Heumez, E. , Pocholle, D. , Pageau, K. , Vanacker, H. , Dubois, F. , Le Gouis, J. *et al* (2006) Combined agronomic and physiological aspects of nitrogen management in wheat highlight a central role for glutamine synthetase. New Phytol. 169, 265–278. 16411930 10.1111/j.1469-8137.2005.01606.x

[pbi13103-bib-0024] Kim, S. , Kang, J.Y. , Cho, D.I. , Park, J.H. and Kim, S.Y. (2004) ABF2, an ABRE‐binding bZIP factor, is an essential component of glucose signaling and its overexpression affects multiple stress tolerance. Plant J. 40, 75–87. 15361142 10.1111/j.1365-313X.2004.02192.x

[pbi13103-bib-0025] Kong, L.G. , Wang, F.H. , Lopez‐bellido, L. , Garcia‐mina, J.M. and Si, J.S. (2013) Agronomic improvements through the genetic and physiological regulation of nitrogen uptake in wheat (*Triticum aestivum* L.). Plant Biotechnol. Rep. 7, 129–139.

[pbi13103-bib-0026] Konishi, M. and Yanagisawa, S. (2013) Arabidopsis NIN‐like transcription factors have a central role in nitrate signalling. Nat. Commun. 4, 1617. 23511481 10.1038/ncomms2621

[pbi13103-bib-0027] Krapp, A. , David, L.C. , Chardin, C. , Girin, T. , Marmagne, A. , Leprince, A.‐S. , Chaillou, S. *et al* (2014) Nitrate transport and signalling in *Arabidopsis* . J. Exp. Bot. 65, 789–798. 24532451 10.1093/jxb/eru001

[pbi13103-bib-0028] Kurai, T. , Wakayama, M. , Abiko, T. , Yanagisawa, S. , Aoki, N. and Ohsugi, R. (2011) Introduction of the *ZmDof1* gene into rice enhances carbon and nitrogen assimilation under low‐nitrogen conditions. Plant Biotechnol. J. 9, 826–837. 21624033 10.1111/j.1467-7652.2011.00592.x

[pbi13103-bib-0029] Li, S. , Tian, Y. , Wu, K. , Ye, Y. , Yu, J. , Zhang, J. , Liu, Q. *et al* (2018) Modulating plant growth‐metabolism coordination for sustainable agriculture. Nature, 560, 595–600. 30111841 10.1038/s41586-018-0415-5 PMC6155485

[pbi13103-bib-0030] Lu, Y.E. , Luo, F. , Yang, M. , Li, X.H. and Lian, X.M. (2011) Suppression of glutamate synthase genes significantly affects carbon and nitrogen metabolism in rice (*Oryza sativa* L.). Sci. China Life Sci. 54, 651–663. 21748588 10.1007/s11427-011-4191-9

[pbi13103-bib-0031] Lu, Y. , Sasaki, Y. , Li, X. , Mori, I.C. , Matsuura, T. , Hirayama, T. , Sato, T. *et al* (2015) ABI1 regulates carbon/N‐nutrient signal transduction independent of ABA biosynthesis and canonical ABA signalling pathways in *Arabidopsis* . J. Exp. Bot. 66, 2763–2771. 25795738 10.1093/jxb/erv086 PMC4986877

[pbi13103-bib-0032] Martin, A. , Lee, J. , Kichey, T. , Gerentes, D. , Zivy, M. , Tatout, C. , Dubois, F. *et al* (2006) Two cytosolic glutamine synthetase isoforms of maize are specifically involved in the control of grain production. Plant Cell, 18, 3252–3274. 17138698 10.1105/tpc.106.042689 PMC1693956

[pbi13103-bib-0033] Masclaux‐Daubresse, C. , Daniel‐Vedele, F. , Dechorgnat, J. , Chardon, F. , Gaufichon, L. and Suzuki, A. (2010) Nitrogen uptake, assimilation and remobilization in plants: challenges for sustainable and productive agriculture. Ann. Bot. 105, 1141–1157. 20299346 10.1093/aob/mcq028 PMC2887065

[pbi13103-bib-0034] Miao, C. , Xiao, L. , Hua, K. , Zou, C. , Zhao, Y. , Bressan, R.A. and Zhu, J.‐K. (2018) Mutations in a subfamily of abscisic acid receptor genes promote rice growth and productivity. Proc. Natl Acad. Sci. USA, 115, 6058–6063. 29784797 10.1073/pnas.1804774115 PMC6003368

[pbi13103-bib-0035] Mokhele, B. , Zhan, X.J. , Yang, G.Z. and Zhang, X.L. (2012) Review: nitrogen assimilation in crop plants and its affecting factors. Can. J. Plant Sci. 92, 399–405.

[pbi13103-bib-0036] Para, A. , Li, Y. , Marshall‐Colón, A. , Varala, K. , Francoeur, N.J. , Moran, T.M. , Edwards, M.B. *et al* (2014) Hit‐and‐run transcriptional control by *bZIP1* mediates rapid nutrient signaling in *Arabidopsis* . Proc. Natl Acad. Sci. USA, 111, 10371–10376. 24958886 10.1073/pnas.1404657111 PMC4104873

[pbi13103-bib-0037] Qu, B. , He, X. , Wang, J. , Zhao, Y. , Teng, W. , Shao, A. , Zhao, X. *et al* (2015) A wheat CCAAT box‐binding transcription factor increases the grain yield of wheat with less fertilizer input. Plant Physiol. 167, 411–423. 25489021 10.1104/pp.114.246959 PMC4326744

[pbi13103-bib-0038] Quraishi, U.M. , Abrouk, M. , Murat, F. , Pont, C. , Foucrier, S. , Desmaizieres, G. , Confolent, C. *et al* (2011) Cross‐genome map based dissection of a N use efficiency ortho‐metaQTL in bread wheat unravels concerted cereal genome evolution. Plant J. 65, 745–756. 21251102 10.1111/j.1365-313X.2010.04461.x

[pbi13103-bib-0039] Raghavendra, A.S. , Gonugunta, V.K. , Christmann, A. and Grill, E. (2010) ABA perception and signalling. Trends Plant Sci. 15, 395–401. 20493758 10.1016/j.tplants.2010.04.006

[pbi13103-bib-0040] Ren, Y. , He, X. , Liu, D. , Li, J. , Zhao, X. , Li, B. , Tong, Y. *et al* (2012) Major quantitative trait loci for seminal root morphology of wheat seedlings. Mol. Breed. 30, 139–148.

[pbi13103-bib-0041] Shan, Q. , Wang, Y. , Li, J. and Gao, C. (2014) Genome editing in rice and wheat using the CRISPR/Cas system. Nat. Protoc. 9, 2395–2410. 25232936 10.1038/nprot.2014.157

[pbi13103-bib-0042] Shao, A. , Ma, W. , Zhao, X. , Hu, M. , He, X. , Teng, W. , Li, H. *et al* (2017) The auxin biosynthetic *TRYPTOPHAN AMINOTRANSFERASE RELATED TaTAR2.1‐3A* increases wheat grain yield. Plant Physiol. 174, 2274–2288. 28626005 10.1104/pp.17.00094 PMC5543937

[pbi13103-bib-0043] Shrawat, A.K. , Carroll, R.T. , DePauw, M. , Taylor, G.J. and Good, A.G. (2008) Genetic engineering of improved N use efficiency in rice by the tissue‐specific expression of alanine aminotransferase. Plant Biotechnol. J. 6, 722–732. 18510577 10.1111/j.1467-7652.2008.00351.x

[pbi13103-bib-0044] Signora, L. , De Smet, I. , Foyer, C.H. and Zhang, H. (2001) ABA plays a central role in mediating the regulatory effects of nitrate on root branching in *Arabidopsis* . Plant J. 28, 655–662. 11851911 10.1046/j.1365-313x.2001.01185.x

[pbi13103-bib-0045] Sun, H. , Qian, Q. , Wu, K. , Luo, J. , Wang, S. , Zhang, C. , Ma, Y. *et al*, *et al* (2014) Heterotrimeric G proteins regulate nitrogen‐use efficiency in rice. Nat. Genet. 46, 652–656. 24777451 10.1038/ng.2958

[pbi13103-bib-0046] Tabuchi, M. , Sugiyama, K. , Ishiyama, K. , Inoue, E. , Sato, T. , Takahashi, H. and Yamaya, T. (2005) Severe reduction in growth rate and grain filling of rice mutants lacking *OsGS1;1*, a cytosolic glutamine synthetase1;1. Plant J. 42, 641–651. 15918879 10.1111/j.1365-313X.2005.02406.x

[pbi13103-bib-0047] Tamura, W. , Hidaka, Y. , Tabuchi, M. , Kojima, S. , Hayakawa, T. , Sato, T. , Obara, M. *et al* (2010) Reverse genetics approach to characterize a function of NADH‐glutamate synthase1 in rice plants. Amino Acids, 39, 1003–1012. 20213442 10.1007/s00726-010-0531-5

[pbi13103-bib-0048] Uno, Y. , Furihata, T. , Abe, H. , Yoshida, R. , Shinozaki, K. and Yamaguchi‐Shinozaki, K. (2000) *Arabidopsis* basic leucine zipper transcription factors involved in an abscisic acid‐dependent signal transduction pathway under drought and high‐salinity conditions. Proc. Natl Acad. Sci. USA, 97, 11632–11637. 11005831 10.1073/pnas.190309197 PMC17252

[pbi13103-bib-0049] Wang, Q. , Nian, J. , Xie, X. , Yu, H. , Zhang, J. , Bai, J. , Dong, G. *et al* (2018a) Genetic variations in *ARE1* mediate grain yield by modulating N utilization in rice. Nat. Commun. 9, 735. 29467406 10.1038/s41467-017-02781-w PMC5821702

[pbi13103-bib-0050] Wang, W. , Hu, B. , Yuan, D. , Liu, Y. , Che, R. , Hu, Y. , Ou, S. *et al* (2018b) Expression of the nitrate transporter gene *OsNRT1.1a/OsNPF6.3* confers high yield and early maturation in rice. Plant Cell, 30, 638–651. 29475937 10.1105/tpc.17.00809 PMC5894839

[pbi13103-bib-0051] Xu, G. , Fan, X. and Miller, A.J. (2012) Plant N assimilation and use efficiency. Annu. Rev. Plant Biol. 63, 153–182. 22224450 10.1146/annurev-arplant-042811-105532

[pbi13103-bib-0052] Yamaya, T. , Obara, M. , Nakajima, H. , Sasaki, S. , Hayakawa, T. and Sato, T. (2002) Genetic manipulation and quantitative‐trait loci mapping for nitrogen recycling in rice. J. Exp. Bot. 53, 917–925. 11912234 10.1093/jexbot/53.370.917

[pbi13103-bib-0053] Yang, X. , Nian, J. , Xie, Q. , Feng, J. , Zhang, F. , Jing, H. , Zhang, J. *et al* (2016) Rice ferredoxin‐dependent glutamate synthase regulates N–carbon metabolomes and is genetically differentiated between japonica and indica subspecies. Mol. Plant, 9, 1520–1534. 27677460 10.1016/j.molp.2016.09.004

[pbi13103-bib-0501] Yanagisawa, S. , Akiyama, A. , Kisaka, H. , Uchimiya, H. and Miwa, T. (2004) Metabolic engineering with *Dof1* transcription factor in plants: Improved nitrogen assimilation and growth under low‐nitrogen conditions. Proc Natl Acad Sci U S A, 101, 7833–7838. 15136740 10.1073/pnas.0402267101 PMC419692

[pbi13103-bib-0054] Yu, L.H. , Wu, J. , Tang, H. , Yuan, Y. , Wang, S.M. , Wang, Y.P. , Zhu, Q.S. *et al* (2016) Overexpression of *Arabidopsis NLP7* improves plant growth under both nitrogen‐limiting and ‐sufficient conditions by enhancing nitrogen and carbon assimilation. Sci. Rep. 6, 27795. 27293103 10.1038/srep27795 PMC4904239

[pbi13103-bib-0055] Zhang, L. , Zhang, L. , Xia, C. , Zhao, G. , Liu, J. , Jia, J. and Kong, X. (2015) A novel wheat bZIP transcription factor, *TabZIP60*, confers multiple abiotic stress tolerances in transgenic *Arabidopsis* . Physiol. Plant. 153, 538–554. 25135325 10.1111/ppl.12261

