# Supplementary material for: Reducing expression of a nitrate‐responsive bZIP transcription factor increases grain yield and N use in wheat
Source: Plant Biotechnol J. 2019 Mar 21;17(9):1823–33. doi: 10.1111/pbi.13103 (PMC6686140; doi:10.1111/pbi.13103)
Supplement: Supplementary file 1 — Figure S1 Phylogenetic analysis of TabZIP60 and the bZIP members from Arabidopsis. Figure S2 Relative expression levels of TabZIP60 and TaNADH‐GOGAT in shoots and roots of their corresponding transgenic lines. Figure S3 Yield‐related traits of the TabZIP60‐6D overexpression lines in the field experiment in the 2015–2016 growing season. Figure S4 N concentrations (%) in aerial organs in TabZIP60 transgenic lines and KN199 during grain filling. Figure S5 Promotor sequence and expression analysis of TaNADH‐GOGAT Figure S6 Relative expression levels of genes involved in N assimilation. Figure S7 Yield‐related traits of the TaNADH‐GOGAT transgenic in the field experiment in the 2015–2016 growing season. [file PBI-17-1823-s002.pdf]

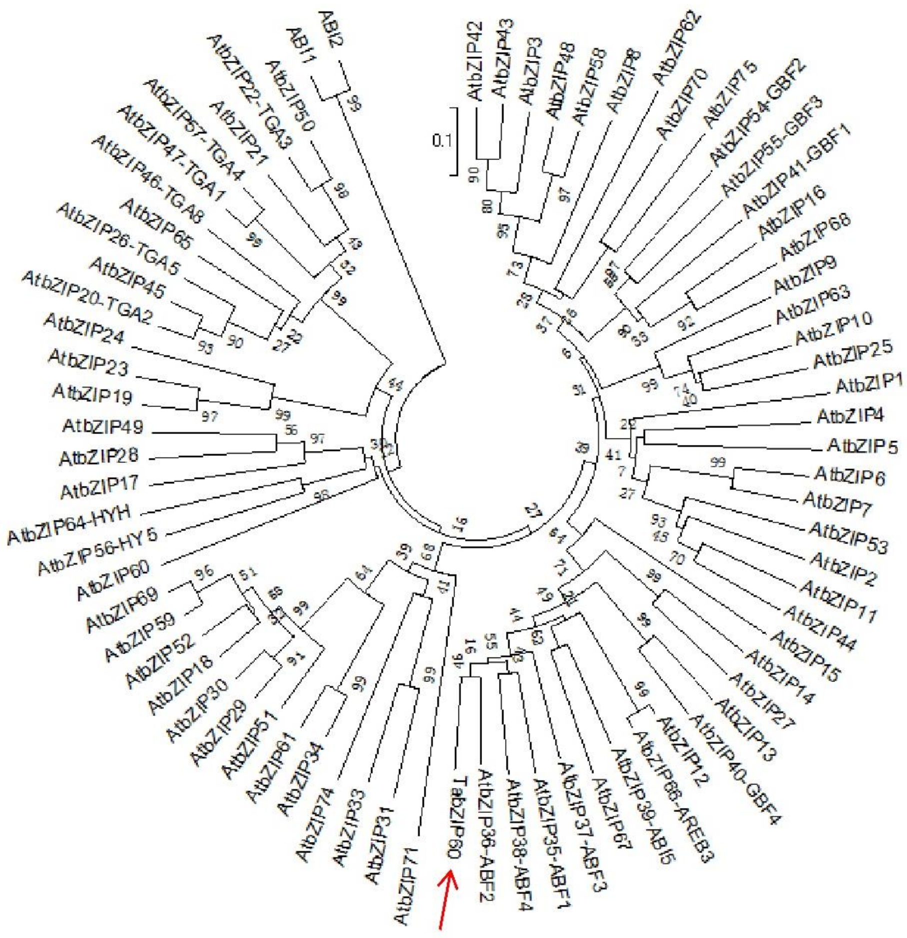


**Supplementary Figure 1.** Phylogenetic analysis of TabZIP60 and the bZIP members from *Arabidopsis*.

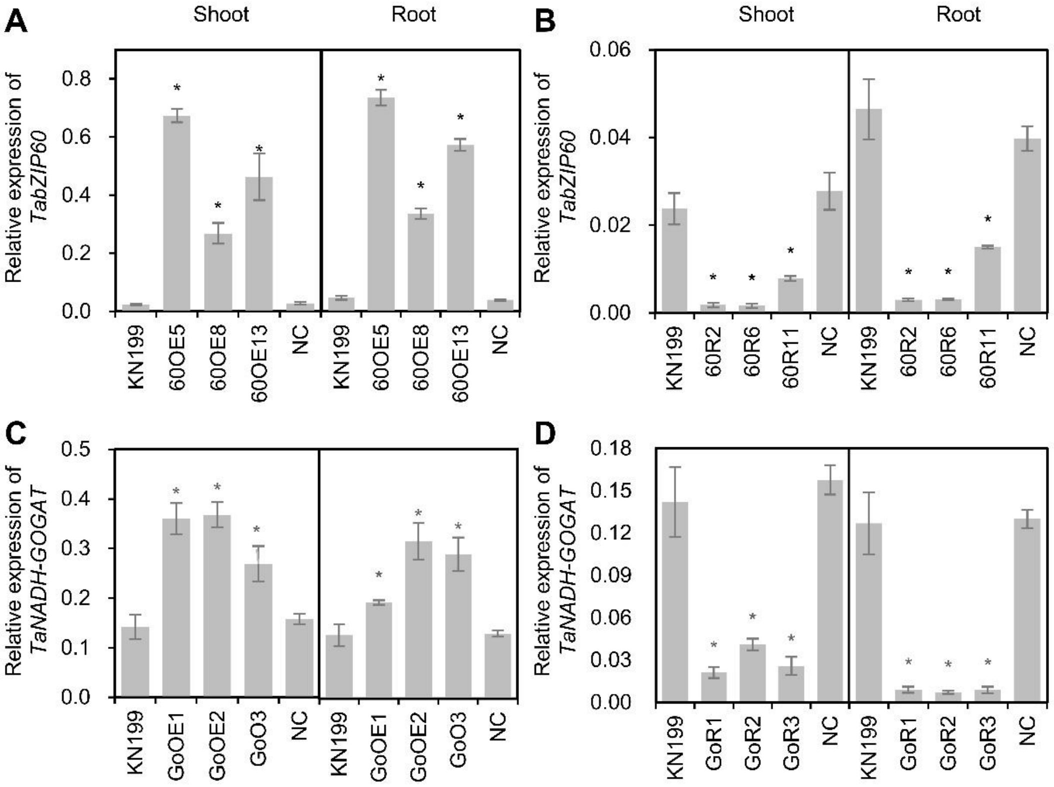


**Supplementary Figure 2.** Relative expression levels of *TabZIP60* and *TaNADH-GOGAT* in shoots and roots of their corresponding transgenic lines. **(A)** Relative expression levels of *TabZIP60* in wild-type, *TabZIP60-6D* overexpression lines and NC. **(B)** Relative expression levels of *TabZIP60* in wild-type, *TabZIP60* RNAi lines and NC. **(C)** Relative expression levels of *TaNADH-GOGAT* in wild-type, *TaNADH-GOGAT-3B* overexpression lines and NC. **(D)** Relative expression levels of *TaNADH-GOGAT* in wild-type, *TaNADH-GOGAT* RNAi lines and NC. Wheat seedlings were grown hydroponically for 14 days. KN199, wild-type; 60OE5, 60OE8 and 60OE13, *TabZIP60-6D* overexpression line; 60R2, 60R6 and 60R11, *TabZIP60* RNAi line; GoOE1, GoOE2 and GoOE3, *TaNADH-GOGAT*-3B overexpression line; GoR1, GoR2 and GoR3, *TaNADH-GOGAT* RNAi line; NC in **(A)** to **(D)** indicates azygous control lines of *TabZIP60-6D* overexpression lines (**A**), *TabZIP60* RNAi lines (**B**), *TaNADH-GOGAT*-3B overexpression lines (**C**), and *TaNADH-GOGAT* RNAi lines (**D**). The relative expression levels were normalized to the expression of *TaACTIN*. Data are means ± SE of three replicates. * indicates the difference between KN199 and transgenic line was signifcant at $P < 0.05$.

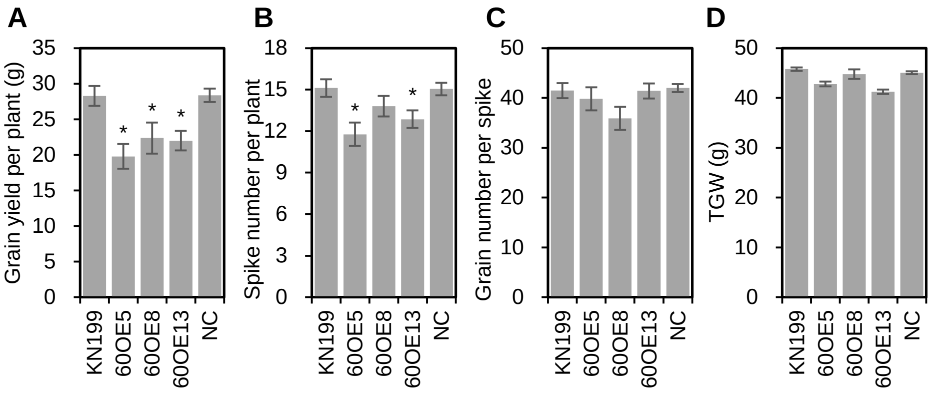


**Supplementary Figure 3**. Yield-related traits of the *TabZIP60-6D* overexpression lines in the field experiment in the 2015–2016 growing season. **(A)** Grain yield. **(B)** Spike number. (**C)** Grain number per spike. (**D)** 1000-grain weight (TGW). KN199, wild-type; 60OE5, 60OE8 and 60OE13, *TabZIP60-6D* overexpression line. NC, azygous control lines. Data are means ± SE of three replicates. * indicates the difference between KN199 and transgenic line was signifcant at $P < 0.05$.

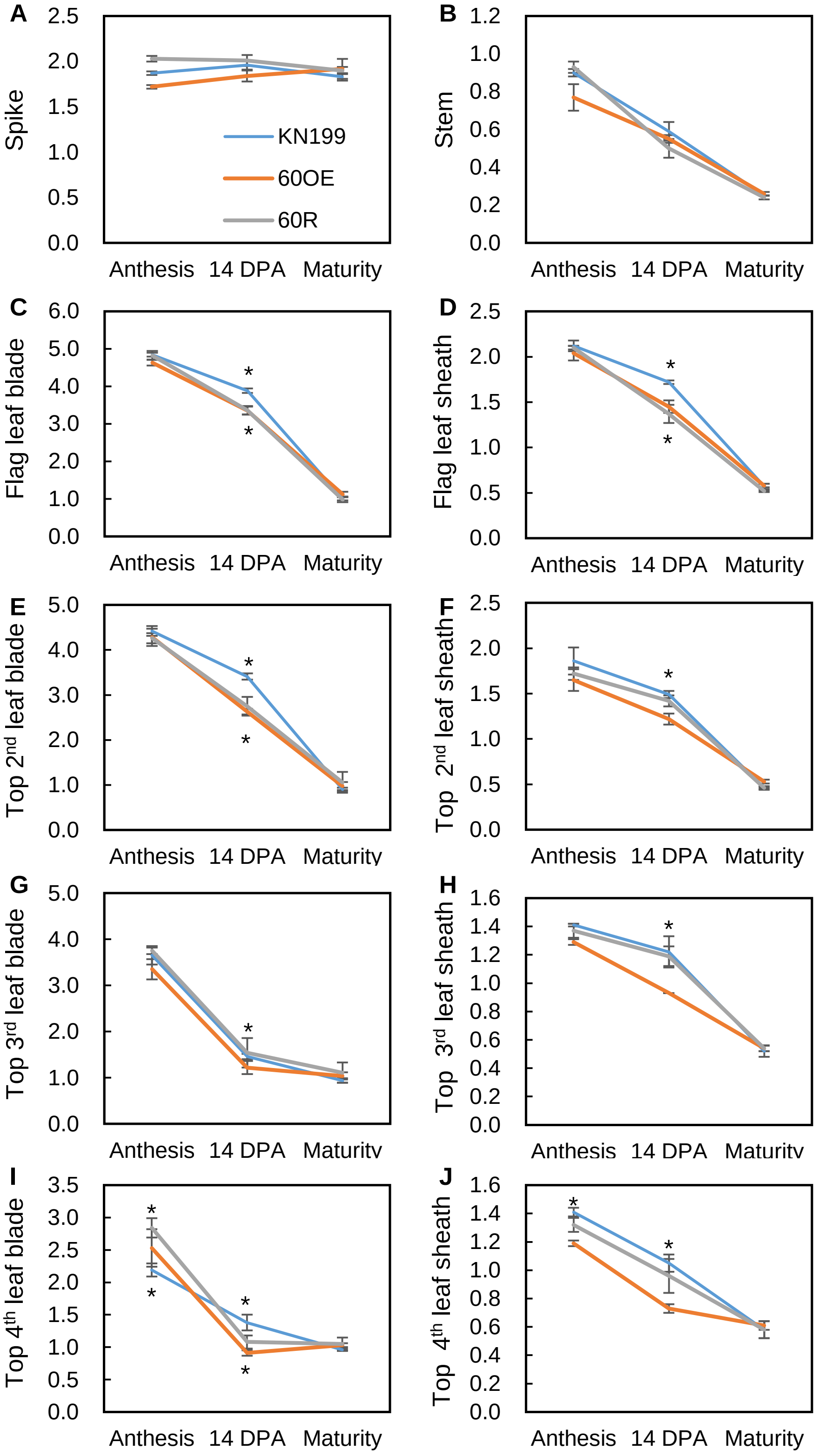


**Supplementary Figure 4**. N concentrations (%) in aerial organs in *TabZIP60* transgenic lines and KN199 during grain filling. (**A**) Spike; (B) Stem; (**C**) Flag leaf blade; (**C**) Flag leaf sheath; (**E**) Top 2nd leaf blade; (F) Top 2nd leaf sheath; (**G**) Top 3rd leaf blade; (**H**) Top 3rd leaf sheath; (**I**) Top 4th leaf blade; (**I**) Top 4th leaf sheath. KN199, wild type; 60OE, *TabZIP60-6D* overexpression lines; 60R, *TabZIP60* RNAi lines. DPA, days post-anthesis. Data for KN199 are mean ± SE (n = 4), Data for 60OE and 60R are mean ± SE of three transgenic lines each with four replications. * above the lines indicates that the difference between KN199 and 60OE line is significant at $P < 0.05$, while that below the lines indicates that the difference between KN199 and 60R line is significant at $P < 0.05$.

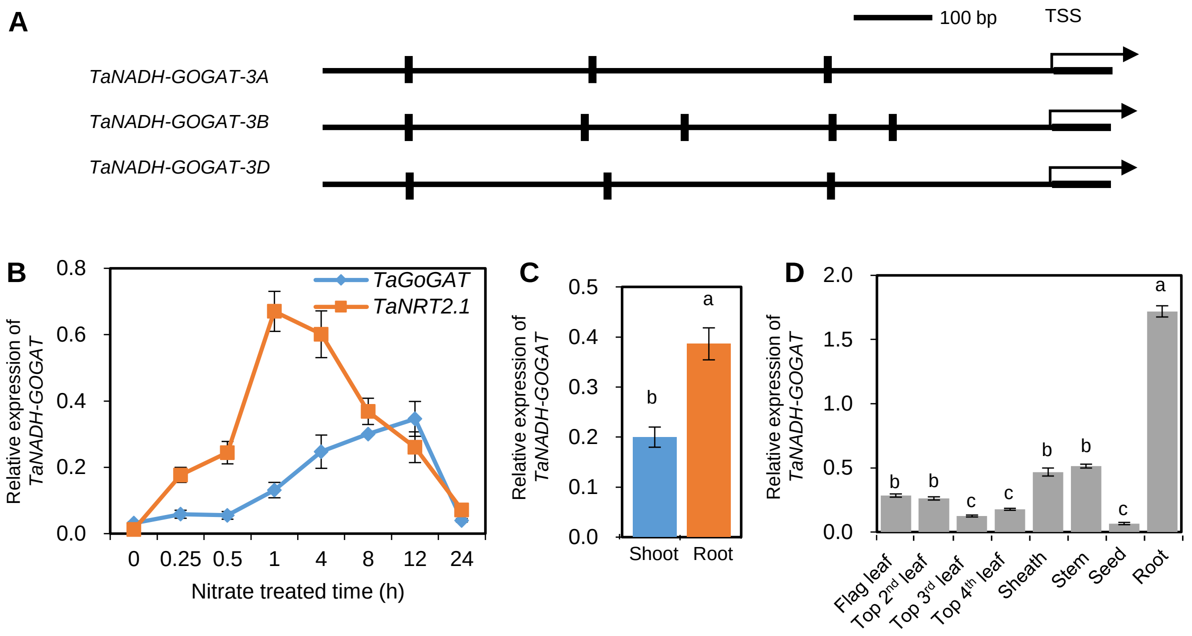


**Supplementary Figure 5.** Promotor sequence and expression analysis of *TaNADH-GOGAT*. **(A)** putative ABRE cis-element in the promoter of *TaNADH-GOGAT* (PlantCARE: bioinformatics.psb.ugent.be/webtools/plantcare/html). Black boxes indicated the putative ABRE cis-element. TSS, transcription start site. **(B)** Expression of *TaNADH-GOGAT* and *TaNRT2.1-6B* in response to nitrate in roots at seedling stage in a hydroponic culture. **(C)** Expression levels of *TaNADH-GOGAT* in shoots and roots at wheat seedling stage in a hydroponic culture. **(D)** Expression of *TaNADH-GOGAT* in different organs of wheat plants at 14 days post anthesis under field conditions. Data are means ± SE (n ≥ 3). Different letters in **(C and D)** indicate statistically significant difference at $P < 0.05$.

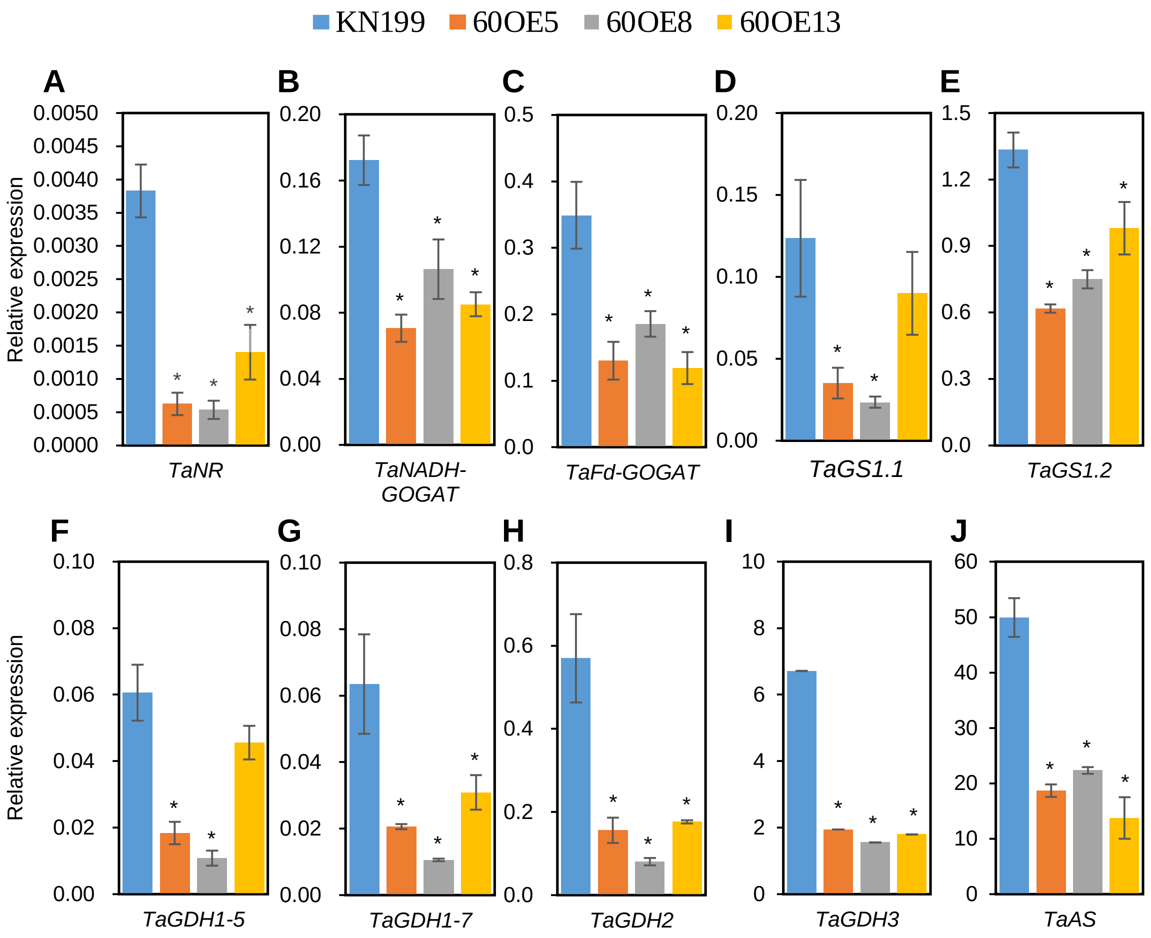


**Supplementary Figure 6.** Relative expression levels of genes involved in N assimilation. **(A)** *TaNR*; **(B)** *TaNADH-GOGAT*; **(C)** *TaFd-GOGAT*; **(D)** *TaGS1.1*; **(E)** *TaGS1.2*; **(F)** *TaGDH1-5*; **(G)** *TaGDH1-7*; **(H)** *TaGDH2*, **(I)** *TaGDH3*; **(J)** *TaAS*. The germinated seedlings were grown in nutrient solution for two weeks, then the roots were collected for gene expression analysis. KN199, wild-type; 60OE5, 60OE8 and 60OE13, *TabZIP60-6D* overexpression line. The relative expression levels were normalized to the expression of *TaACTIN*. The data are expressed as the mean ± S.E. of three replicates. * indicates that the difference between KN199 and the transgenic line is significant at $P < 0.05$.

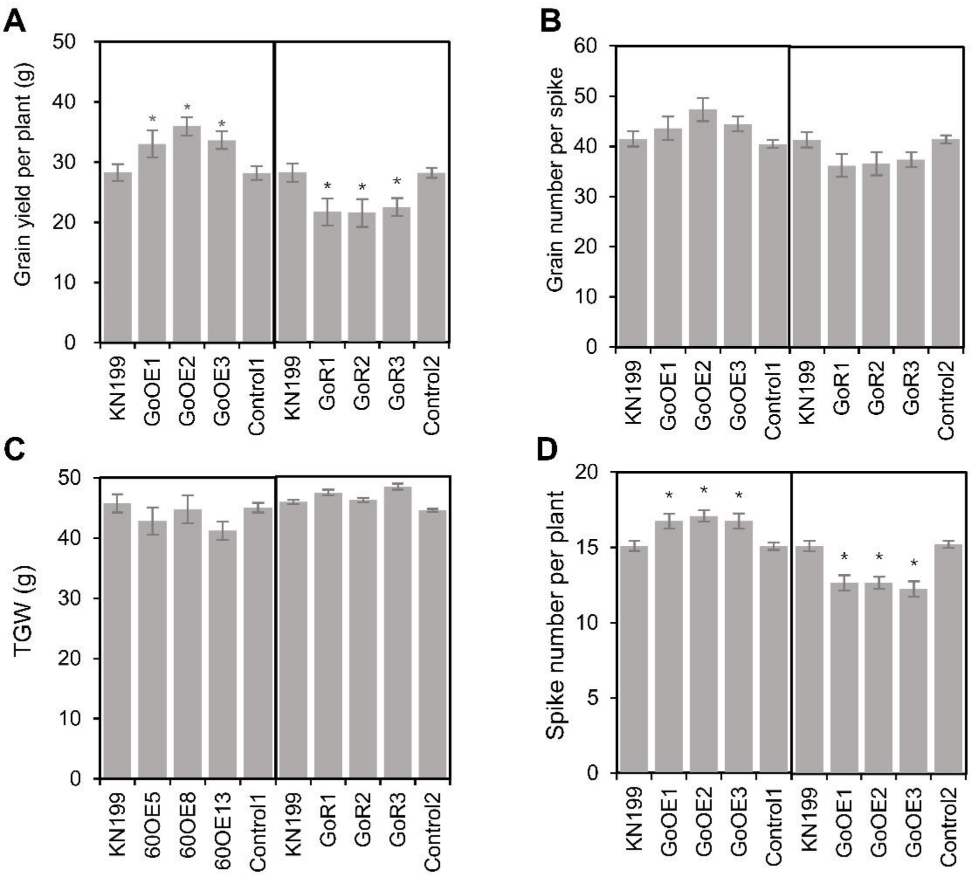


**Supplementary Figure 7.** Yield-related traits of the *TaNADH-GOGAT* transgenic in the field experiment in the 2015–2016 growing season. **(A)** Grain yield. **(B)** Grain number per spike. **(C)** 1000-grain weight (TGW). **(D)** Spike number per plant. KN199, wild-type; GoOE1, GoOE2 and GoOE3, *TaNADH-GOGAT*-3B overexpression line; GoR1, GoR2 and GoR3, *TaNADH-GOGAT* RNAi line; Control1 and 2, azygous control. Data are means ± SE, n = 3. * indicates the difference between KN199 and transgenic line was signifcant at $P < 0.05$.
